# Overuse of Computed Tomography Pulmonary Angiography and Low Utilization of Clinical Prediction Rules in Suspected Pulmonary Embolism Patients at a Regional Australian Hospital

**DOI:** 10.3390/healthcare12020278

**Published:** 2024-01-22

**Authors:** Li Ning Chean, Clement Tan, Matthew I. Hiskens, Marie Rattenbury, Prahalath Sundaram, Jithmy Perara, Karen Smith, Pranav Kumar

**Affiliations:** 1 Mackay Base Hospital, Mackay 4740, Australiaclement.tan@health.qld.gov.au (C.T.);; 2 College of Medicine and Dentistry, James Cook University, Mackay 4740, Australia

**Keywords:** pulmonary embolism, computed tomography pulmonary angiography, clinical prediction rule, venous thromboembolism

## Abstract

A pulmonary embolism (PE) is an obstruction in the pulmonary arterial system and may include non-specific signs and symptoms. Clinical prediction rules (CPRs) assess the pretest probability (PTP) of a PE to prevent the overuse of computed tomography pulmonary angiography (CTPA). CTPA overuse results in patient harm and health system waste. This study aimed to evaluate CTPA usage in an Australian regional hospital through analyzing CTPA encounters. A retrospective chart analysis was undertaken of 100 CTPAs conducted at an Australian regional hospital from April to May 2023. Analysis was undertaken for parameters including risk factors, signs and symptoms, investigations, and the use of CPRs. Overall, 86% of patients had signs and/or symptoms of a PE within a week of examination, and 6% of the population had signs of deep vein thrombosis. More than half of the population had no risk factors, while the most prevalent risk factors were a recent history of immobilization/trauma and/or having surgery that required general anesthesia in the last 4 weeks. The most common co-morbidity was chronic lung disease (11%). For the pre-test diagnostic workup, the ECG was the most ordered investigation. The Wells’ score was used at 10%, while most patients did not have any CPRs applied. The prevalence of PEs discovered on CTPAs was 9%. CPRs were under-utilized in this Australian regional hospital. The D-dimers for ruling out subjects with low PTP derived from CPRs were also underused. This led to the inappropriate overordering of CTPAs, resulting in negative implications for patients and unnecessary costs to the health system.

## 1. Introduction

A pulmonary embolism (PE) is not uncommon and can often be fatal. A PE is defined as an obstruction in the pulmonary arterial system from either a clot, fat, air, or tumor sources. A PE can have significant mortality impacts and is currently the third highest cause of cardiovascular death worldwide [1]. The majority of PEs originate from Deep Vein Thrombosis (DVT) in the lower extremities. Both PEs and DVTs fall under the umbrella term of Venous Thromboembolism (VTE) [2]. The pathophysiology of VTE can be described simply by the elements of Virchow’s triad-hypercoagulability, stasis, and endothelial damage [1].

The extent of hemodynamic compromise due to a PE can be related to the degree of obstruction of the vasculature and impact to the right ventricle (RV). The resulting response can range from RV dilation to obstructive shock and death. Given the pathophysiology and resultant effects of a PE, signs and symptoms can be non-specific, adding to the challenge of diagnosing a PE [1,3]. Symptoms can range from hemoptysis, unilateral lower limb pain/swelling, tachycardia, chest pain, shortness of breath, and hypoxia. Each of these symptoms carry a wide differential diagnosis for clinicians, and most patients with these symptoms will not have a PE [1,2,3,4,5]. In addition, it is critical to also identify potential risk factors for VTE when considering this potential diagnosis. Risk factors commonly associated with VTE include hormone use (as either replacement or contraception), immobilization (including recent surgery, trauma, or prolonged bed rest), and a previous history of VTE and malignancy [2,6]. Furthermore, individuals with additional co-morbidities, such as chronic lung disease, heart failure, and thrombophilia, face an additional level of risk for potential VTE [2,7].

The clinical prediction rules (CPRs) for a PE help to calculate the pre-test probability (PTP) scores to assist clinicians in objectively determining the clinical likelihood of a VTE diagnosis. CPRs consider risk factors that are identified through a patient’s clinical history [3,4,8], and are recommended to help rule out a PE before progressing to computed tomography pulmonary angiography (CTPA) for a definitive diagnosis. Frequently used CPRs include the Pulmonary Embolism Rule-out Criterion rule (PERC), Geneva score, and Wells’ score. The utilization of these tools allows categorization and provides guidance for evidence-based practice, thus reducing the potential for over-investigation and the inappropriate use of imaging modalities [6,9].

CTPA is a validated imaging modality for the diagnosis of a PE and is regarded as the gold standard for diagnosing a PE due to its high sensitivity and specificity [6,7,10]. Not only is CTPA commonly available, but various studies have found CTPA to be more accurate in PE detection in comparison to the ventilation-perfusion (V/Q) scan [3,10,11]. CTPA has the added benefit of the prognostication of PE-related impacts (such as providing information regarding RV strain and dysfunction), and it can assist in diagnosing other non-VTE associated etiologies for the cause of a patient’s symptom/s [1,2].

Due to the immense advantages of CTPA, there is emerging evidence indicating overreliance as a diagnostic tool and overuse in the clinical setting [10,11]. The key reasons for overuse include the availability of scanners, ease of ordering processes, poor understanding of the clinical consequences of excessive imaging use, and fear of clinicians and patients in missing a diagnosis of a PE [11,12]. It is important to note that this overuse of CTPAs is not without adverse outcomes, and can involve renal toxicity from the use of iodine-based contrast media and radiation exposure to vulnerable groups such as young children, young women, and pregnant women [6,9,10]. Additionally, as CTPAs can detect anything from hemodynamically unstable PEs to clinically insignificant PEs, and the treatment of PEs can carry significant risks, a risk-benefit analysis must be individualized to each patient prior to commencing treatment [5,11]. The overuse of CTPA also carries a financial burden and wasted resources within the healthcare system is a major concern [9].

CPRs function as validated algorithms/guidelines on a pre-test diagnostic workup that is available to guide clinicians [8]. However, there are not many published studies/audits that confirm that these guidelines are adhered to in real-world practice. Therefore, the aim of this study was to assess the diagnostic practices relating to suspected PEs and the usage of CTPAs in an Australian regional hospital by analyzing and describing the following: 1. pre-test CTPA/PE diagnostic workup, and 2. resultant findings of these CTPAs.

## 2. Materials and Methods

This study was a retrospective chart review of patients who underwent a CTPA at Mackay Base Hospital, a regional Australian hospital. The study was approved by the Townsville Hospital and Health Service Human Research Ethics Committee (EX/2023/QTHS/100999). The STROBE guidelines for reporting observational studies were used [13].

### 2.1. Data Collection

Integrated Electronic Medical Records (ieMR) is the medical documentation system in place within the hospital facility at which this study was conducted. ieMR was accessed for the collection of the clinical data used in this study. The audit was undertaken for 100 consecutive patients over the course of a 30-day period from the month of April to May 2023. Data were collected to assess basic demographic information, clinically significant points on history, and key PE workup findings along with the presence of the use of CPRs for a PE, if any. Any key quantifiable investigative findings are based on the ranges provided by Pathology Queensland. Of note, the D-dimer value is based on age-adjusted levels. The imaging and pathology data queried were only retrieved for the period during which the patient received their CTPAs. All variables collected with the analysis tool can be found in Table 1. The inclusion criteria specified inpatients and Emergency Department patients over the age of 18 who had a CTPA. The exclusion criteria included patients who had CTPAs completed as outpatients and who had been asked to represent to the hospital for further management. Patients who had two CTPAs completed within the study period only had their first CTPA included in the study. Each encounter was uniquely reviewed by one of five authors (LC, CT, MR, PS, JP) independently, and the data collected were entered into the confidential data collection tool. All authors were trained in the use of the data collection tool prior to commencement, and the data were assessed at the completion of collection, with any disagreements between case reviewers resolved via discussions. To reduce potential sources of bias, none of the case reviewers were involved in the original clinical decisions or the care of the patients involved in the study.

### 2.2. Data Analysis

Data were analyzed using the IBM Statistical Package for the Social Sciences 26.0 software (IMB Corp, Armonk, NY, USA). Demographic data are presented as frequencies and/or proportions, with continuous data reported as the mean and standard deviation. Pearson Chi-Square tests were performed to compare the parameters between patients with and without a PE. Clinical prediction rules were assessed through the proportions of the true positive and negative findings yielded within the study. The D-dimer was assessed for sensitivity, specificity, the positive predictive value (PPV), and the negative predictive value (NPV).

## 3. Results

**Figure 1:**
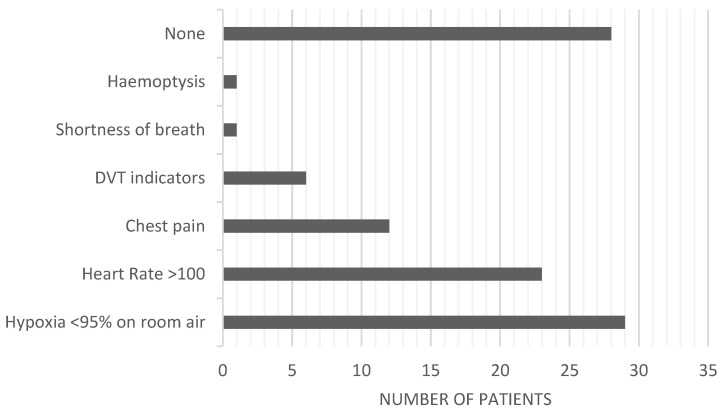
Signs and symptoms of patients assessed with suspected PE.

Table 2 presents patient demographics, risk factors, and co-morbidities. Importantly, more than half of the study population did not have a risk factor for a PE, and 77% did not have a co-morbidity. Figure 1 illustrates the prevalence of signs and symptoms in the patient cohort. Of the 100 subjects, 29 subjects presented with hypoxia < 95% on room air, followed by 23 subjects who had tachycardia and 12 subjects who had chest pain which prompted clinicians to suspect a PE. Of note, only 6% of the study population had signs of DVT, which is an essential sign used in most CPRs for a PE.

Table 3 shows the blood assays and investigations that were ordered prior to proceeding for a CTPA. The selected findings from these results include only 1/9 elevated BNP findings, which were associated with the presence of a PE, and BNP was normal in 4/9 PE-positive subjects. While this test showed significant differences between groups, this was related to the high proportion of patients who did not have an ordered test. The ECG was by far the most ordered investigation when a PE is suspected in this study, with 85% of subjects receiving an ECG prior to CTPA. However, of the nine subjects with a positive PE, two had a normal ECG.

Nine patients out of one hundred were confirmed as positive for a PE (Table 3). Table 4 shows the details for the patients with positive CTPA findings. Seven patients showed a segmental PE, with one suggesting evidence of right heart strain and the remaining two showing a subsegmental PE on the CTPAs. As mentioned in the section of data collection, patients who received two CTPA studies within our study period only had their first CTPA study included in this study. However, for disclosure, four patients out of one hundred had a repeat CTPA completed an average of five days after their first due to persistent or slow improving symptomatology.

CPRs were assessed in a small number of patients in this study. The diagnostic performance of a D-dimer assay was 100% sensitivity, 5.3% specificity, a positive predictive value (PPV) of 7.7%, and a negative predictive value (NPV) of 100%. The PERC rule was applied in 3% of subjects. The Wells’ score was calculated in 10% of subjects while the Geneva score was utilized in 2% of individuals. As a result, the PERC rule did not identify any of the 9 PEs with 3/91 false positives. The Geneva score identified 1/9 PEs with 1/91 false positive. The Wells’ score identified one PE with 9/91 false positives. The remaining individuals did not receive any recorded forms of CPR use. It is important to note that despite only nine positive PEs being found on CTPA, a total of 14 patients received an initial treatment for a PE. The most frequently prescribed treatment for a PE in these subjects was the introduction of a novel anticoagulant (9/14), followed by low molecular weight heparin (4/14), and the use of thrombolytics (1/14).4. 

## 4. Discussion

We conducted a retrospective review of 100 patients who received a CTPA to assess if CTPAs are overordered within this Australian regional hospital. We included an evaluation of the subjects’ relevant risk factors, signs and symptoms, investigation findings, and the use of CPRs as pretest diagnostic workups.

The ECG findings of S1Q3T3 found in our study support the finding of another study that S1Q3T3 is of high specificity but of a limited sensitivity [14]. The arterial blood gases completed can show the partial pressure of arterial oxygen content, which would help calculate the resultant A-a gradient. The A-a gradient, if normal, is otherwise not reliable in excluding PEs in patients with or without cardiopulmonary disease [15]. Hence, we may conclude that the two arterial blood gases completed amongst the total study population can be regarded as clinically insignificant in this context.

The nature of the PERC rule as a criterion for ruling out a PE has been reported as a CPR of high sensitivity and NPV, but with a low specificity in a meta-analysis [16]. In our study, the PERC rule yielded three false positives in the three times that it was used, which is in keeping with the reported low specificity. However, this could have been due to the small number of PERC rule usage in the population studied. The Geneva score was not shown to be a viable alternative CPR, as a significant number of PEs did not progress to CTPAs in a separate study [17]. There were 1/91 false positives yielded in this study; however, due to the small number of its uses, it can be challenging to reach a definitive conclusion on its use.

The NPV of 100% for the D-dimer drawn from this study reflects the findings of Wells and colleagues in the study validating their CPR tool [4]. They demonstrated a D-dimer NPV of 99%, which was used in combination with their Wells’ score clinical algorithm to exclude the need for lung imaging in 44% to 47% of subjects with a suspected pulmonary embolism [4]. The sensitivity of a D-dimer was 100% in this study, which is higher than a previous study which observed a sensitivity of 87% with the Clearview Simplify D-dimer assay [18]. This performance in NPV and sensitivity allows the confident exclusion of disease and can hence be carefully applied here to omit unnecessary imaging, thus reducing patient exposure to unnecessary radiation and unnecessary costs for the hospital and health service [10,11].

The proper documentation of CPRs is key to highlighting the PTP to allow for the proper utilization of a D-dimer assay. Furthermore, a Wells’ score of 0 should prompt the clinician to reconsider ordering imaging for a PE given the low prevalence of PEs in low PTP groups when the Wells’ score was appropriately applied [18,19]. Another study conducted in an Australian tertiary hospital reinforces this through the demonstration of a significant reduction in the number of CTPAs ordered when the documentation of the PTP of the Wells’ score and D-dimer results in appropriate groups was made compulsory [9].

The 2019 European Society of Cardiology (ESC) Guidelines for the Diagnosis and Management of Acute PE state that the Initial risk stratification (Class I) is to be conducted in all individuals with a suspected or confirmed PE [3,7]. This will involve the measurement of various clinical parameters including the simplified Pulmonary Embolism Severity Index (PESI) score, hemodynamic, RV function, and elevated biomarkers such as BNP and troponin.

The inappropriate ordering of CTPA imposes a significant cost to the healthcare system, as the cost of a single CTPA is $530.65 as per the Australian Medicare rebate and the cost is estimated to be higher when staff wages for conducting and reporting the imaging are taken into play [10]. It was estimated that the total financial cost of CTPA per diagnosis of a PE increased from A$3450 to A$21803 with its use while the PE prevalence in the low PTP group decreased from 6% to 1% [18]. With the increased global utilization of CT-scanners and the escalation of costs, private and public institutions will pass along higher costs to funders including governments and insurance providers. An illustration of the cost savings that can be achieved is provided in the study by Ong and colleagues (2012), who reported a saving of $61 710 by performing 121 fewer CTPA in a seven-month period following an intervention emphasizing the use of clinical predication rules [9]. Furthermore, the nationwide emergence of the heavy locum workforce may contribute to further challenges in addressing the inappropriate ordering of CTPA; this is because locum workforces do not have key performance indicators, such as cost-related ones in this instance, stipulated within their contract, but rather, only stipulated working timeframes [20]. However, there is common acknowledgement regarding the limitations of our study. These include the small number of audited patients, the retrospective nature of the study, and the possibility that this study may not be generalized in the context of it being conducted only within Mackay Base Hospital.

## 5. Conclusions

This study has identified the low utilization of CPRs, namely the Wells’ score, PERC rule, and Geneva score, in our regional hospital. In addition, the D-dimer as an investigation for ruling out subjects with low PTP in this regional hospital was also under-utilized. This indicates a general low threshold for ordering CTPAs causing inappropriate overuse, and hence, the over testing of PEs leading only to a low proportion of PE diagnoses within our study population.

Future studies including a thorough cost analysis must be conducted to look at the cost-effectiveness of a qualitative D-dimer and CTPAs when these clinical rules are applied appropriately. It is recommended that there should be more consistency in working up patients with suspected PEs, including establishing a clear clinical pathway that would incorporate compulsory documentation of these clinical prediction rules followed by a possible justification of D-dimers to warrant a CTPA. The appropriate and adequate use of these clinical prediction rules would thereby unequivocally lead to significant cost-effectiveness within the health service and would not expose patients to unnecessary radiation that will have long term harmful health effects.

## Figures and Tables

**Table 1:** Analysis tool used for data collection containing basic demographics, significant clinical history, key investigation findings, and clinical prediction rules.

Parameters	Options
1: Age	
2: Gender	Male Female
3: Symptom Duration	Within a week More than a week
4: Signs and Symptoms	Shortness of breath Chest pain DVT signs (unilateral leg pain/swelling) Hypoxia (<95% on room air) Heart rate > 100 Haemoptysis None of the above
5: Risk Factors	Immobilization/Trauma in the last 3 days or surgery requiring general anesthesia in the last 4 weeks Previous DVT/PE Hormone use (including oral contraceptive pill, hormone replacement therapy, estrogen hormone, and male testosterone) Malignancy (active on treatment in the last 6 months or palliative) None of the above
6: Co-Morbidities	Thrombophilia Chronic lung disease Heart failure with treatment None of the above
7: D-Dimer (Age-Adjusted)	Elevated Not elevated Not ordered
8: Troponin	Elevated Not elevated Not ordered
9. Arterial Blood Gas (ABG)	Venous blood gas completed instead ABG with normal PaCO2 ABG with elevated PaCO2 Not ordered
10: B-type Natriuretic Peptide (BNP)	Elevated Normal Not ordered
11. Electrocardiogram	Yes (ECG findings) - Normal ECG - S1 Q3 T3 pattern - Sinus tachycardia - Right bundle branch block - Right axis deviation - Atrial fibrillation - None of the above 2. No
12. Chest X-ray	Yes - Normal - Prominent pulmonary artery - Pleural effusion - None of the above 2. No
13. PERC Rule	Yes No
14. Geneva Score	Yes No
15. Wells’ Score	Yes No
16. CTPA Findings	
17. Initial Treatment for PE	NIL Novel anticoagulants Low molecular weight heparin Thrombolysis

**Table 2:** Patient demographics, risk factors, and co-morbidities.

	Total (*n* = 100)
Age, yrs, median (IQR)	62 (46–72)
Female gender	63 (63%)
Symptom duration	
- Within a week	86 (86%)
- More than a week	14 (14%)
Risk factors	
- Immobilization/trauma	20 (20%)
- Previous DVT/PE	8 (8%)
- Hormone use	2 (2%)
- Malignancy	13 (13%)
- None of the above	57 (57%)
Co-morbidities	
- Thrombophilia	5 (5%)
- Chronic lung disease	11 (11%)
- Heart failure with treatment	7 (7%)
- None of the above	77 (77%)

**Table 3:** Clinical investigation findings in patients with and without PE.

Variable	No PE	PE Present	χ^2^	*p*
D-Dimer			0.38	0.83
- Elevated	36	3		
- Not elevated	2	0		
- Not ordered	53	6		
Troponin			0.19	0.91
- Elevated	15	1		
- Not elevated	49	5		
- Not ordered	27	3		
Arterial Blood Gas			3.97	0.265
- Venous blood gas instead	39	6		
- ABG with normal PaCO2	3	1		
- ABG with elevated PaCO2	4	0		
- Not ordered	45	2		
B-Type Natriuretic Peptide			8.82	0.012
- Elevated	10	1		
- Normal	9	4		
- Not ordered	72	4		
Electrocardiogram			6.36	0.384
- Normal ECG	37	2		
- S1 Q3 T3 pattern	2	0		
- Sinus tachycardia	17	2		
- Right bundle branch block	4	0		
- Right axis deviation	2	1		
- Atrial fibrillation	7	0		
- None of the above	11	0		
- Not ordered	11	4		
Chest X-ray			3.57	0.312
- Normal	19	1		
- Prominent pulmonary artery	2	0		
- Pleural effusion	8	3		
- None of the above	14	2		
- Not ordered	48	3		
Initial Treatment for PE			63.37	<0.001
- Nil	86	0		
- Novel anticoagulants	3	6		
- Low molecular weight heparin	2	2		
- Thrombolysis	0	1		

**Table 4:** Clinical findings for the nine patients positive for PE.

Age	Signs and Symptoms	Risk Factors	Co-Morbidities	D-Dimer	Troponin	ABG	BNP	ECG	CXR	CPR	CTPA Findings
37	Hypoxia < 95% on room air	None	None	Not ordered	Not elevated	VBG	Normal	Sinus tachycardia	No	Not calculated	Segmental and subsegmental PE
42	Heart rate > 100	None	None	Not ordered	Not elevated	VBG	Normal	Sinus tachycardia	Normal	Not calculated	Segmental PE
58	Hypoxia < 95% on room air	None	None	Elevated	Not ordered	Not ordered	Normal	Normal	Pleural effusion	Not calculated	Segmental PE
60	Hypoxia < 95% on room air	None	Chronic lung disease	Not ordered	Not elevated	VBG	Not ordered	Right axis deviation	Normal	Not calculated	Linear filling defect involving the anterior subsegmental branches of the right upper lobe pulmonary artery, likely suggestive of pulmonary embolism
62	Heart rate > 100	Immobilization/trauma	None	Not ordered	Not ordered	ABG with PCO2 normal	Not ordered	Normal	Pleural effusion	Not calculated	Two small bilateral non-occlusive segmental pulmonary emboli
67	Heart rate > 100	Malignancy	None	Not ordered	Not ordered	VBG	Not ordered	Not ordered	Pleural effusion	Geneva and Well’s score	Positive for PE. Prominent pulmonary embolus at the origin of the pulmonary trunk. Further thromboembolic disease in right lower lobe
84	Hypoxia < 95% on room air	None	None	Elevated	Not elevated	VBG	Not ordered	Normal	Not ordered	Not calculated	Several segmental PEs
85	None	Immobilization/trauma	Chronic lung disease	Elevated	Elevated	VBG	Elevated	Not ordered	None of the above	Not calculated	Bilateral PE with evidence of right heart dysfunction
86	DVT signs	Immobilization/trauma	None of the above	Not ordered	Not elevated	Not ordered	Normal	Normal	Not ordered	Not calculated	Bilateral pulmonary emboli—w/saddle at R pulmonary trunk—R and L segmental and subsegmental involvement

## Data Availability

The datasets analyzed through this study are available from the corresponding author upon reasonable request.
